# A Novel Biochemical Route for Fuels and Chemicals Production from Cellulosic Biomass

**DOI:** 10.1371/journal.pone.0031693

**Published:** 2012-02-23

**Authors:** Zhiliang Fan, Weihua Wu, Amanda Hildebrand, Takao Kasuga, Ruifu Zhang, Xiaochao Xiong

**Affiliations:** 1 Biological and Agricultural Engineering Department, University of California Davis, Davis, California, United States of America; 2 Department of Plant Pathology, University of California Davis, Davis, California, United States of America; 3 Agricultural Research Service, United States Department of Agriculture, Davis, California, United States of America; 4 College of Resources and Environmental Sciences, Nanjing Agricultural University, Nanjing, Jiangshu, People's Republic of China; University of Nottingham, United Kingdom

## Abstract

The conventional biochemical platform featuring enzymatic hydrolysis involves five key steps: pretreatment, cellulase production, enzymatic hydrolysis, fermentation, and product recovery. Sugars are produced as reactive intermediates for subsequent fermentation to fuels and chemicals. Herein, an alternative biochemical route is proposed. Pretreatment, enzymatic hydrolysis and cellulase production is consolidated into one single step, referred to as consolidated aerobic processing, and sugar aldonates are produced as the reactive intermediates for biofuels production by fermentation. In this study, we demonstrate the viability of consolidation of the enzymatic hydrolysis and cellulase production steps in the new route using *Neurospora crassa* as the model microorganism and the conversion of cellulose to ethanol as the model system. We intended to prove the two hypotheses: 1) cellulose can be directed to produce cellobionate by reducing β-glucosidase production and by enhancing cellobiose dehydrogenase production; and 2) both of the two hydrolysis products of cellobionate—glucose and gluconate—can be used as carbon sources for ethanol and other chemical production. Our results showed that knocking out multiple copies of β-glucosidase genes led to cellobionate production from cellulose, without jeopardizing the cellulose hydrolysis rate. Simulating cellobiose dehydrogenase over-expression by addition of exogenous cellobiose dehydrogenase led to more cellobionate production. Both of the two hydrolysis products of cellobionate: glucose and gluconate can be used by *Escherichia coli* KO 11 for efficient ethanol production. They were utilized simultaneously in glucose and gluconate co-fermentation. Gluconate was used even faster than glucose. The results support the viability of the two hypotheses that lay the foundation for the proposed new route.

## Introduction

**Figure 1:**
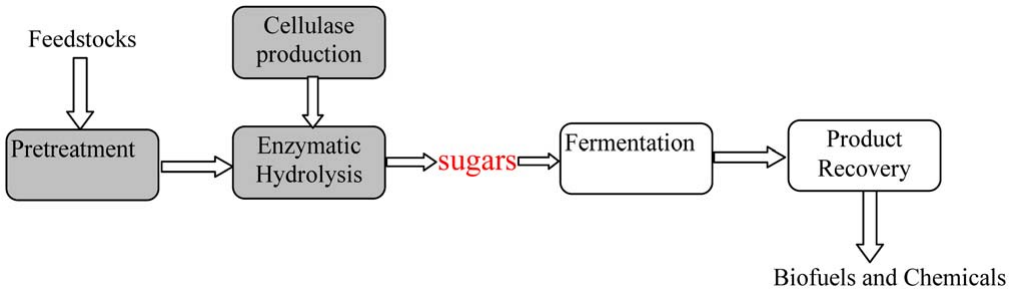
Conventional biochemical platform for biofuels and chemicals production [3], [4].

Cellulosic biomass is a sustainable source for organic fuels, chemicals, and materials; and is available at low cost and in large abundance [1], [2], [3]. The central obstacle impeding the widespread utilization of cellulosic biomass is the absence of a low-cost processing technology [3]. The conventional biochemical platform featuring enzymatic hydrolysis involves five key steps: pretreatment, cellulase production, enzymatic hydrolysis, fermentation, and product recovery, as shown in Figure 1. Sugars are produced as reactive intermediates for subsequent fermentation to fuels and chemicals. The steps involved in overcoming the recalcitrance of cellulosic biomass (making sugars)—pretreatment, cellulase production and enzymatic hydrolysis—are the three costliest steps in the entire process. Lowering the processing cost of these three steps is essential in order to make cellulosic biorefineries economically viable [3], [4], [5].

**Figure 2:**
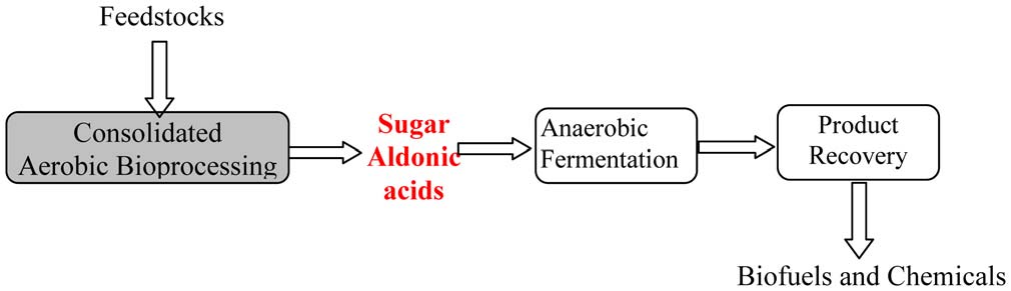
The proposed new route for biofuels and chemicals production.

One strategy to reduce the processing cost is through process consolidation. Herein, we propose an alternative route for fuels and chemical production, which combines cellulase production and enzymatic hydrolysis into a single biological step and produces sugar aldonates instead of sugars as the reactive intermediates. As shown in Figure 2, this new route will utilize microorganism(s) that secrete all of the enzymes needed to solubilize lignin and hydrolyze cellulose and hemicellulose to the resulting sugars, despite the presence of associated lignin. Once formed, the majority of the sugars will be oxidized to the corresponding sugar aldonic acids, such as cellobionic acid or xylobionic acid, thereby preventing sugar utilization by microorganisms. A small fraction of the sugars will be available to support cell growth and enzyme production. In the second step, sugar aldonates (instead of sugars) will be utilized as the reactive intermediates for production of biofuels and other chemicals.

Lignocellulolytic fungi have remarkable potential for cellulosic biomass pretreatment, cellulase production, and direct enzymatic hydrolysis. Biological pretreatment is an attractive alternative to thermal chemical pretreatment due to its low energy requirements, mild reaction conditions, and environmental benefits [4], [6]. Most biological pretreatment studies have been focused on use of lignocellulolytic microorganisms such as brown-, white- and soft-rot fungi or cellulolytic bacteria to selectively remove lignin in lignocellulosic materials, either to increase the digestibility of lignocellulose or as a pretreatment reagent for bio-pulping [6], [7], [8], [9]. Of these options, white rot fungi are the preferred microorganisms for the pretreatment tasks due to their ability to degrade lignin. However, two factors impede the practical application of biological pretreatment: carbohydrate loss during the treatment process and the slow reaction rate. To solve the carbon loss problem, alternative strategies have been proposed to selectively delignify lignin and suppress carbon-degrading enzymes [10], [11], [12], [13]. Research has been conducted to select for naturally-occurring white rot fungi that preferentially attack lignin [14], [15], [16], selecting cellulase-less mutants [8], [17], [18], or repressing enzymes that degrade wood carbohydrates [19]. Limited success was achieved in all of these cases. Because lignin does not provide a carbon source for microbial growth of white rot fungi, repressing carbohydrate hydrolysis enzymes limits the reactivity of these fungi and leads to even longer processing times. There is no efficient strategy to tackle the slow growth problem of the biological pretreatment, and the genetic modification of white rot fungi is rather difficult. Lignocellulolytic fungi remain the main workhorse for cellulase production in industry. However, they have not been directly used in conversion of cellulose to sugars, also due to the fact that the fungi consume sugars for their own growth, which leads to severe carbohydrate loss during the process.

In the proposed new route, “consolidated aerobic bioprocessing,” intends to consolidate the pretreatment, cellulase production, and enzymatic hydrolysis steps in the conventional platform into a single microbiological step. Hence, we need to solve the same two problems associated with the utilization of fungi in biological pretreatment and direct enzymatic hydrolysis: slow growth rates and carbohydrate loss. The strategy we adopt to tackle the slow growth problem is to use the fast-growing fungus such as *Neurospora crassa*, which produces powerful cellulases, hemicellulases, and a variety of oxidases and laccases that are involved in phenol degradation, and potentially in lignin modification [20], [21], [22]. It is a genetically-tractable microorganism and tools for its genetic engineering are readily accessible [23], [24], [25]. Although *N. crassa* does not produce as powerful ligninases as white rot fungi, it has the potential for improvement of its lignin degrading ability by engineering the lignin degrading enzymes. Moreover, we will adopt a metabolic engineering strategy to solve the carbohydrate loss problem. Specifically, carbohydrate loss will be controlled by modifying the fungus' metabolic pathway so that sugar aldonates instead of sugars will be produced from cellulose. In a separate step, sugar aldonates will be converted to fuels and chemicals by fermentation.

In this study, we aim to demonstrate the feasibility of consolidation of the enzymatic hydrolysis and cellulase production steps in the new route using *N. crassa* as the model microorganism and conversion of cellulose to ethanol as the model conversion system. The feasibility of consolidation of the cellulase production and enzymatic hydrolysis steps is based on the following two hypotheses:

Hypothesis 1: Cellulose can be directed to cellobionic acid production by reducing β-glucosidase production and by enhancing cellobiose dehydrogenase production.

**Figure 3:**
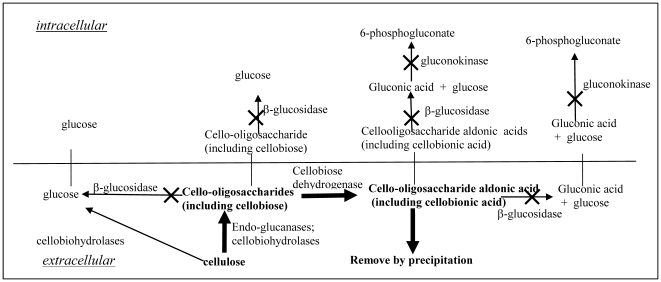
The mechanism of cellulose degradation by some cellulolytic fungi.

Figure 3 shows the cellulose degradation mechanism for some cellulolytic microorganisms, including white rot fungi, brown rot fungi, and *Neurospora* [14], [26], [27], [28], [29], [30]. Cellulose is hydrolyzed by endoglucanases and exoglucanases to cello-oligosaccharides, with cellobiose as the main component. Cellobiose and other cello-oligosaccharides are hydrolyzed by extracellular β-glucosidase (BGL) to form glucose or will be dehydrogenated by cellobiose dehydrogenase (CDH) to produce cellobionolactone, which reacts spontaneously with water to form cellobionic acid [31], [32], [33]. Cellobiose and other cello-oligosaccharides can also be transported intracellularly and be hydrolyzed by intracellular BGL to form glucose. Cellobionic acid and cello-oligosaccharide aldonic acids also can be hydrolyzed by intracellular or extracellular BGL to form glucose and gluconic acid, both of which can be metabolized by fungi [34].

As shown in Figure 3, cellobiose and cello-oligosaccharides can be diverted to the production of cellobionic acid and other cello-oligosaccharide aldonic acids if all the BGL productions are blocked. In practice, some BGL activity must be maintained for cellulase induction and glucose generation. However, it is still possible to divert the majority of the carbon flow toward aldonic acid formation by over-expressing CDH, and lowering the activity of BGL. The added advantage is that organic acids are produced as products and methodologies for organic acid recovery such as calcium hydroxide precipitation can be adopted to remove them from the fermentation broth continuously so that they will not accumulate at high enough concentrations if they are inhibitory to the enzymes and the microorganisms [35]. However, detailed studies on the inhibitory effect of aldonate are still to be carried out Meanwhile, blocking gluconic acid utilization by inactivating the gluconokinase gene (*gnk*), which encodes the enzyme that is responsible for the first step in the gluconic acid utilization pathway, will improve the cellobionic acid yield from cellulose. As shown in Figure 3, gluconic acid is the end product of the reaction of cellobionic acid hydrolysis. Gluconic acid was found to be a non-competitive product inhibitor to the BGL from *Aspergillus niger* and its presence could prevent any noticeable hydrolysis of cellobionic acid by BGL [36].

Hypothesis 2: Both of the two hydrolysis products of cellobionate—glucose and gluconate—can be used as carbon sources for ethanol production.

Cellobionate and other cello-oligosaccharide aldonic acids can be easily converted to glucose and gluconate by adding exogenous β-glucosidase or by engineering in the fermenting microorganisms the ability to produce β-glucosidase in the subsequent fermentation step. Glucose is easily utilized by various microorganisms to produce ethanol and other chemicals. Theoretically, sugar aldonates can be utilized via the Entner-Doudoroff pathway to produce a wide variety of fermentation products including ethanol. From one mole gluconic acid, 1.5 moles of ethanol, 0.5 mole of acetic acid and 1.5 moles of ATP will be generated. If starting from the substrate cellobionic acid (Equation 2), 1.75 moles of ethanol, 0.25 mole of acetate and 1.75 mole of ATP are produced from sugar acids on a per glucose equivalent basis.
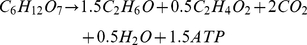
(1)

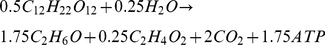
(2)


However, there was no experimental verification of these theoretical predictions found in literature.

In this study, experiments were designed to demonstrate the feasibility of the two hypotheses which lay the foundation for the consolidation of the enzymatic hydrolysis and cellulase production steps in the proposed new route.

## Results and Discussion

### Cellobionate production by the multiple *bgl* knockout strain with or without exogenous CDH addition

**Figure 4:**
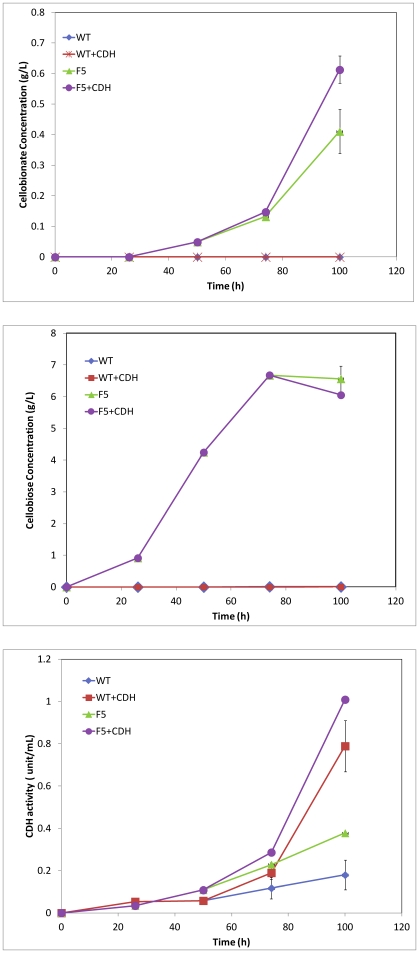
The cellobionate, cellobionate and CDH production by wild type and strain F5 with or without CDH addition.

The hextuple *bgl* knockout strain F5 was able to produce about 0.4 g/L and 6.5 g/L cellobionate and cellobiose from cellulose, while the wild type strain did not produce any detectable amount of cellobionate or cellobiose. As shown in Figure 4, addition of the exogenous CDH increased the concentration of cellobionate produced by strain F5 from 0.4 g/L to 0.6 g/L. Strain F5 converted cellulose faster and produced more CDH and less cell mass as compared to the wild type. As shown in Table 1, the cellulose conversion achieved with F5 was 64%, as compared to that of wild type (55%). The mycelium yields from Avicel were about 22% and 52% for strain F5 and for the wild type, respectively.. As shown in Figure 3, the highest CDH detected in the broth of F5 was 0.79 IU/L, as compared to 0.18 IU/L for the wild type. About 52% of Avicel was converted to cellobiose and cellobionate by strain F5, while no Avicel has been diverted to cellobiose and cellobionate production by the wild type strain.

**Table 1:** Summary of cellulose conversion and mycelium cell mass production.

	Starting Avicel (g)	Residual Avicel (g)	Cellulose Conversion (%)	Mycelium produced (g)	Yield of cellobiose and cellobionate from consumed Avicel (mol/mol×100%)	Yield of mycelium mass from consumed Avicel (g/g×100%)
wild type	1.0	0.47±0.05	53±5%	0.28±0.02	0	52±5%
wild type+CDH	1.0	0.40±0.03	60±3%	0.31±0.04	0	51±7%
F5	1.0	0.36±0.01	64±1%	0.14±0.02	52±7%	22±2%
F5+CDH	1.0	0.35±0.0002	65±0.02%	0.16±0.01	49±2%	24±1%

Our results clearly showed that cellobionate was produced from cellulose by a mutant *N. crassa* strain with six out of seven copies of *bgl* knocked out. Simulating CDH over-expression by adding exogenous CDH produced more cellobionate. Substantial amount of cellobiose (about 6 g/L) was accumulating in the broth, which is an indication that the conversion of cellobiose to cellobionate is limiting in the system. It seemed that knocking out six copies of *bgl* not only did not affect the cellulose utilization by this strain.

### Gluconate can be utilized as the substrate for ethanol production by recombinant *E. coli* KO 11 strain

**Figure 5:**
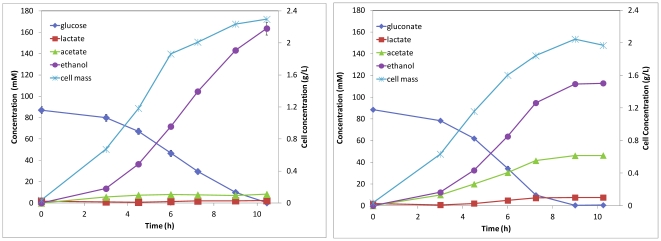
Ethanol production from glucose and gluconate by *E. coli* KO11.

Glucose and gluconate were investigated as the sole carbon source for ethanol production by *E. coli* KO 11, which was engineered for efficient ethanol production from sugars [37]. Both glucose and gluconate were used for ethanol production. As shown in Figure 5, ethanol was the main fermentation product and very little acetate and lactate was produced when glucose was utilized as the substrate. About 163.6 mM of ethanol was produced from 87.1 mM glucose, reaching 94% of the theoretical yield. When gluconate was utilized as the substrate, ethanol and acetate were found to be the two main products, along with a small amount of lactate. About 113 mM ethanol and 46.3 mM of acetate were produced from 88.5 mM of gluconate. The stoichiometry of ethanol and acetic acid produced from gluconate follows Equation 1. Ethanol yield reached 85% of the theoretical yield, while acetate reached 105% of the theoretical yield. The gluconate was metabolized faster than glucose.

**Figure 6:**
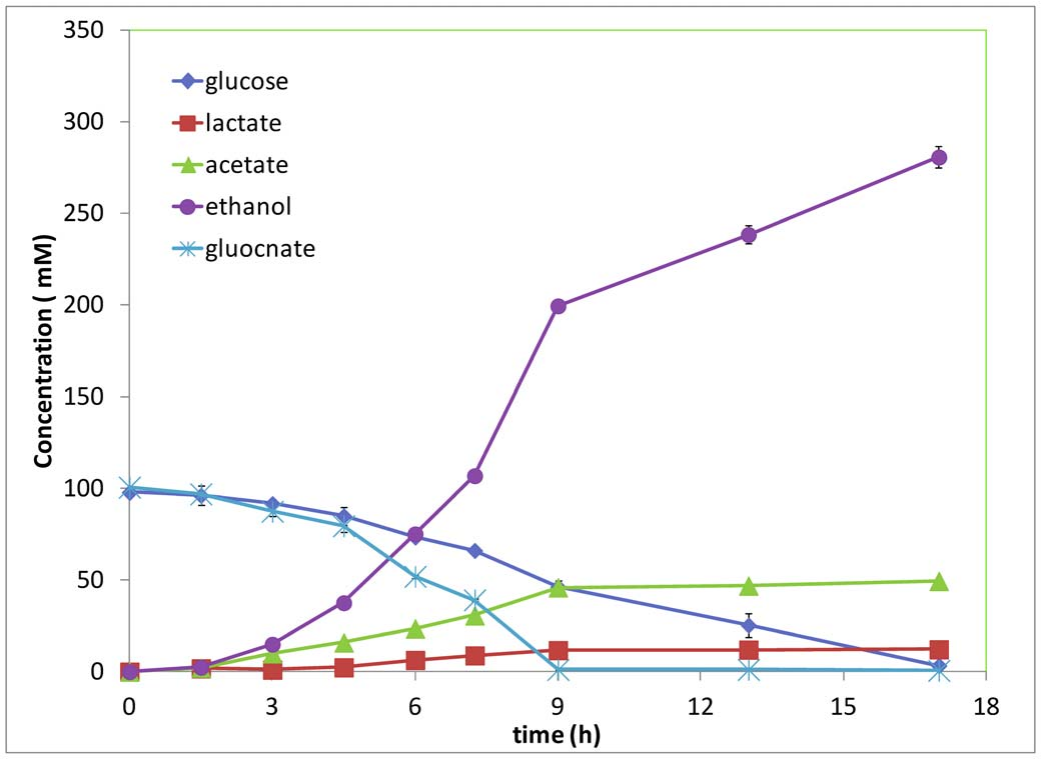
Ethanol and acetic acid production from glucose and gluconate co-fermentation.

Since one mole of glucose and one mole of gluconate will be generated from cellobionate hydrolysis, we also studied the glucose and gluconate co-utilization by *E coli*. KO11. Glucose and gluconate co-fermentation was conducted starting with about 100 mM of glucose and 100 mM of gluconate. It was found that glucose and gluconate were utilized simultaneously. Ethanol and acetate were the two main products and the amounts produced follow the stoichiometry of equation 2. Produced ethanol and acetate reached about 80.7% and 99.6% of the theoretical yields, respectively. Gluconate was, again, found to be utilized faster than glucose (Figure 6).

*E. coli* was found to be able to metabolize gluconate aerobically [38], [39]. According to our knowledge, our study is the first to report that gluconate is able to be metabolized for the production of fermentation products by *E. coli* under anaerobic conditions. Gluconate seems to be an excellent substrate for fermentation. It was utilized faster than glucose when they were used separately or in a co-culture. The same trend was found in *E. coli* JM 101 when glucose and gluconate were used as the carbon source in an aerobic culture [39]. The reason why gluconate is utilized faster than glucose is still to be elucidated. It is likely due to the different efficiency of their transporters and glucose and gluconate are transported by different transporters in *E. coli* [39], [40], [41]. Glucose and gluconate were also found to be utilized by *E. coli* KO 11 simultaneously when both of them were supplied as the carbon source, which indicated that the catabolite repression effect of glucose on gluconate was not obvious.

The proposed new route represents a substantial different route for fuels and chemicals production from cellulosic biomass. Sugar aldonates were produced as the reactive intermediates for the subsequent fermentation to fuels and chemicals. Since sugar aldonates are more reduced than glucose, a small amount of acetate has to be produced along with glucose, which led to lower yield of ethanol. However, the loss due to the production of acetate is relative small. Taking the production of cellobionic acid as an example, the yield of ethanol from cellobionic acid is about 87.5% of that from glucose on per glucose equivalent basis and the ratio of acetate produced verses ethanol is one to seven. The ethanol yield on a per glucose basis will increase when the chain length of cellooligosaccharide aldonates increases. Since the amount of acetate is relatively small, it is probably not economical to recover it as an individual product from the fermentation broth. However, in a biorefinery, most of the acetate will remain in the wastewater and can be easily converted to methane in the wastewater treatment step and the energy contained in acetate can be recovered.

### Conclusion

Our results showed that knocking out multiple copies of β-glucosidase genes led to cellobionate production from cellulose, without jeopardizing the cellulose hydrolysis rate. Simulating cellobiose dehydrogenase over-expression by addition of exogenous cellobiose dehydrogenase led to more cellobionate production. Both of the two hydrolysis products of cellobionate: glucose and gluconate can be used by *Escherichia coli* KO 11 for efficient ethanol production. They were utilized simultaneously in glucose and gluconate co-fermentation. Gluconate was used even faster than glucose. Our preliminary results support the viability of the two hypotheses that lay the foundation for the proposed new route.

## Materials and Methods

### Microbial strains and culture conditions

All the single *bgl* knockout strains were from Fungal Genetic Stock Center (FGSC). The spores were stocked in glycerol at −80°C. *E. coli* KO11 (ATCC 29191) was purchased from ATCC and was stocked in the −80°C.

### Construction of hextuple *bgl* knock out strain

Multiple *bgl* knockout strains were constructed through genetic crossing following a standard mating protocol [42]. Knockout strains were selected using a PCR-genotyping method. Since each single *bgl* knockout mutant from the FGSC contains a hygromycin resistance gene (*hph^r^*) inserted within a specific *bgl* locus, reverse primers were designed based on the *hph^r^* open reading frame and forward primers were designed based on the flanking sequence of the knockout loci. The double knockout strains should produce two PCR products corresponding to two replaced genes. Two single knockout strains were crossed to generate the double knockout strain. Quadruple knockout strains were constructed by crossing two double knockout strains. The procedures were repeated until obtaining hextuple knockout strains. The *bgl* loci have been successfully knocked out in a mutant strain designated F5, and the diagnostic primer pairs for PCR genotyping are listed in Table 2.

**Table 2:** The primers used for PCR geno-typing.

Locus Number	Primers	Sequence
NCU00130	left primer	5′-ACACACCCAAGCACAAACGAACA-3′
NCU04952	left primer	5′-GAAAGCCACAACATCTCGTCCAC-3′
NCU05577	left primer	5′-TAATTCGTTCACCCACTCTGCCA-3′
NCU07487	left primer	5′-AAGCAAGCTCTCAACACCTCTCG-3′
NCU08755	left primer	5′-TTTCTCGCGACCTTCTCTCTTCC-3′
NCU03641	left primer	5′-TATCGGAAAAGACCTGGCAACCT-3′
hgh	right primer	5′-AGAGCTTGGTTGACGGCAATTTC-3′

### Preparation of crude CDH and CDH activity assay

The crude CDH was produced from a recombinant strain of *Pichia pastoris*, which heterologously expresses CDH from *N. crassa* following the production and concentration protocols as described by Zhang et al [43]. CDH activity was measured using 2,6-dichlorophenol-indophenol (DCPIP) as the electron acceptor as described in Zhang et al [43]. One international unit (IU) was equivalent to the reduction of 1 µmol of DCPIP per min at 30°C.

### Cellobionate production by F5 and the wild type strains

The wild type and the hextuple *bgl* knockout strain F5 were cultured in Vogel's medium with Avicel and glucose supplied at 20 g L^−1^ and 0.8 g L^−1^, respectively, as the carbon source. Fermentation was initiated by inoculating 10-day old conidia to the flasks at the initial concentration of 10^6^ mL^−1^. The fermentation was carried on a rotary shaker at 200 rpm and at 27°C for 100 hours with the light on. Samples were taken at various time intervals to monitor the concentrations of cellobiose, glucose, cellobionate, and the activity of CDH in the broth. Exogenous CDH was added at 0.07 IU mL^−1^ at 48 hours and 0.27 IU mL^−1^ at 74 hours to selected bottles. After 100 hours of culture, the residues were filtered and dried at 72°C for two days. The residual mycelium dry weight was indirectly measured by measuring the nitrogen content in the dried samples using a LECO TruSpec CHN elemental determinator (St. Joseph, MI, USA). The residual Avicel was calculated from the weight difference between the residual sample and the mycelium dry weight.

### Anaerobic fermentation experiment

Anaerobic fermentations were carried out in 200 mL serum bottles with a working volume of 100 mL and a N_2_ gas phase. Luria Bertani (LB) broth with glucose or gluconate or both as the carbon source was used as the culturing medium. The pH was adjusted to 7.0. The batch experiments were initiated by inoculating the 1% of *E coli* KO 11 inoculums and incubating on a rotary shaker at 200 rpm and at 37°C. Samples were taken at various time intervals. The concentrations of glucose, gluconate, acetate, lactate, and ethanol were analyzed by HPLC. The cell concentration was measured by spectrophotometer at 600 nm. The cell mass concentration was calculated based on a calibration curve correlating cell concentration with absorbance.

### Sample analysis

The concentrations of sugars, organic acids, and aldonates in the broth were analyzed using a Shimazdu HPLC system equipped with RID detector. The concentrations of sugars and sugar aldonates in the samples were determined using an Aminex HPX-87C column (Bio-Rad) at 80°C. The effluent was 4 mM CaCl_2_ at a flow rate of 0.5 ml min^−1^. The acetic acid, lactic acid and ethanol were determined by an ICSep ION-300 column from Transgenomic (San Jose, CA, USA) at 60°C. The effluent was 5 mM of H_2_SO_4_ at a flow rate of 0.6 ml min^−1^.

### Statistical analysis

All the experiments were conducted with duplicate samples. SAS JMP (Cary, NC, USA) was used for analyzing the results.
